# Supplementary material for: Does the site of research evidence generation impact on its translation to clinical practice? A protocol paper
Source: PLoS One. 2024 Dec 13;19(12):e0314956. doi: 10.1371/journal.pone.0314956 (PMC11643258; doi:10.1371/journal.pone.0314956)
Supplement: S1 Appendix — (DOCX) [file pone.0314956.s002.docx]

Site: Ward: Total number of beds: Date: Time:

| **Falls Prevention Strategy Category** | **Falls Prevention Strategy** | **Tally (count the number of times each strategy is observed in the ward)** | **Total** |
| --- | --- | --- | --- |
| Environment – all beds in ward regardless of patient presence | Falls risk-related signage and education materials **[please add rows as necessary for the different types at your health service]** |  |  |
|  | **Mobilisation alarm in place (may or may not be active) [ESSENTIAL – please add rows as necessary for the different types at your health service]** |  |  |
|  | Family in room |  |  |
|  | Companion/sitter |  |  |
|  | Regular bed in low position |  |  |
|  | Floor line bed in floor position |  |  |
|  | Floor mat by bed |  |  |
| When patient is present and alert | Patient visible from outside room |  |  |
|  | Number of patients present for audit |  |  |
|  | Call bell within reach |  |  |
|  | Table within reach |  |  |
|  | Mobile/Bedside Phone within reach |  |  |
|  | Bedside commode/urinal |  |  |
|  | **Mobilisation alarm in place and active [ESSENTIAL – please add rows as necessary for the different types at your health service]** |  |  |
|  | Ambulatory aid accessible to patient (within reach without taking a step) |  |  |
|  | Physical restraint in place |  |  |
|  | Non-slip footwear being worn |  |  |
|  | Non-slip socks being worn |  |  |
| Additional comments (e.g. room closed for maintenance on DATE) |  | | |

***Troubleshooting:***

- ***Do I document the presence of a family member in the room if they are only visiting temporarily?***
  - ***Yes, document anything you observe during the ward visit.***
- ***If the patient is in the bathroom or resting, do I still need to find out if they’re wearing non-slip footwear?***
  - ***No, there is no need to disturb patients to document data. Only note down what you can observe during the visit.***
