# Supplementary material for: Does the site of research evidence generation impact on its translation to clinical practice? A protocol paper
Source: PLoS One. 2024 Dec 13;19(12):e0314956. doi: 10.1371/journal.pone.0314956 (PMC11643258; doi:10.1371/journal.pone.0314956)
Supplement: S1 Protocol — (DOCX) [file pone.0314956.s004.docx]

**Using disinvestment to investigate the effectiveness of mobilisation alarms in a stepped-wedge randomised trial design in tertiary hospitals**

Project Description and Protocol

Version 1.4, 15 September 2023

**Project Team Roles & Responsibilities**

There will be 7 chief investigators (CI) and 2 research fellows contributing to the proposed study, their roles and responsibilities are shown in the table below.

| **Name, Position, Affiliation** | **Roles and Responsibilities** |
| --- | --- |
| **Chief Investigators** | |
| Professor Terrence Haines  Head of School of Primary and Allied Health Care, Faculty of Medicine, Nursing and Health Sciences, Monash University | In this project, Prof. Haines will be responsible for project leadership, recruitment of staff to project roles, performance management of all project staff, ensuring that reports for the Independent Data Monitoring Committee are prepared on the required monthly schedule, liaison with executive members from participating sites, development of the data analysis and economic analysis plans, manuscript preparation, and delivery specific actions within the knowledge translation plan. |
| Professor Ronald Shorr  Director of the Geriatric Research Education and Clinical Center, Malcolm Randall Veterans Affairs Medical Center  Research professor, Department of Epidemiology, University of Florida  Director of the Training and Research Academy in Clinical and Translational Science, University of Florida | Prof. Shorr will be responsible for development of data collection methodologies for capturing the use of bed alarms along with other important falls prevention measures using observation methods. He will assist in development of methods for collection of falls data. He will contribute to development of the statistical analysis plan, preparation of manuscripts, reports and other materials for use in knowledge translation activities for use in Australia and internationally. |
| Professor Mari Botti  Alfred Deakin Professor, School of Nursing and Midwifery, Faculty of Health, Deakin University  Chair of Nursing, Epworth HealthCare | Prof. Botti will be a consultant for the ethical approval processes necessary for this study, and contribute towards strategies to facilitate research implementation within the health services that will be recruited for the project. She will also contribute to the qualitative methodology for this study. |
| Doctor Natasha Brusco  Senior Research Officer, School of Primary and Allied Health Care, Faculty of Medicine, Nursing and Health Sciences, Monash University | Dr. Brusco will be responsible for developing the methodology for the health economics component of the study. Following data collection, she will also be responsible for analysing and interpreting the results from this aspect of the study. |
| Associate Professor Elizabeth O’Brien  Director of Research, School of Primary and Allied Health Care, Faculty of Medicine, Nursing and Health Sciences, Monash University | For this project, Dr. O’Brien will contribute towards the methodological design of the qualitative component of this study. She will direct the collection and analysis of qualitative data, specifically regarding staff reactions to change in practice. |
| Associate Professor Bernice Redley  Associate Professor of Nursing (Monash Health), School of Nursing and Midwifery, Faculty of Health, Deakin University | Dr. Redley will contribute to the development of ward data collection procedures, particularly observational methods for collection of data related to other falls prevention practices. She will contribute to manuscript preparation and site recruitment. |
| Doctor Kelly-Ann Bowles  Senior Lecturer and Director of Research, School of Primary and Allied Health Care, Faculty of Medicine, Nursing and Health Sciences, Monash University | Dr. Bowles’ role in this project will be to lead the data extraction plan and oversee preparation of data reports for the Independent Data Monitoring Committee. She will assist in development of the statistical analysis plan and contribute to manuscript preparation. |
| **Associate Investigators** | |
| Adjunct Associate Professor Leanne Boyd | Associate investigators will act as advisors to project development and liaisons for individual study sites (health services). |
| Associate Professor Peter Hunter |  |
| Professor Alison Hutchinson |  |
| Ms. Joanna Jellett |  |
| Dr. Samantha Sevenhuysen |  |
| Ms. Debra Mitchell |  |
| Doctor Melisaa Raymond |  |
| Ms. Kate Steen |  |
| Ms. Melinda Webb St-Mart |  |
| Associate Professor Philip Russo |  |
| **Professional Research Staff** | |
| Doctor Dai (Debbie) Pu  Research Fellow, School of Primary and Allied Health Care, Faculty of Medicine, Nursing and Health Sciences, Monash University | Dr. Pu will be a full-time manager for this project. She will co-ordinate project activities and committees, facilitate stakeholder participation in project planning and dissemination, lead identification of potential project wards for the matched control arm, lead communications with ward staff and management, initiate ethics applications, oversee ward eligibility testing, develop standardised operating procedures and monitor data collection as the study progresses, facilitate extraction of data from hospital data collection systems at participating sites, develop the written and video results summary for part 1, and manuscript preparation throughout the project. |

**Resources**

Funding has been secured for this project from the National Health and Medical Research Council’s (NHMRC) Ideas Grants (Project code: APP1186185). This funding will support the employment of professional research staff and 12 research assistants who will collect data offsite, and costs associated with the administrative processes for project approval and data extraction.

Contents

[Background 5](#_Toc74921867)

[Research Aims 6](#_Toc74921868)

[Expected Outcomes 6](#_Toc74921869)

[Project Design 8](#_Toc74921870)

[Research Project Setting 8](#_Toc74921871)

[Methodological Approach 8](#_Toc74921872)

[Participants 9](#_Toc74921873)

[Inclusion criteria 9](#_Toc74921874)

[Exclusion criteria 10](#_Toc74921875)

[Participant Recruitment and Time Frame 10](#_Toc74921876)

[Health Services 10](#_Toc74921877)

[Hospital Wards 11](#_Toc74921878)

[Hospital Staff 11](#_Toc74921879)

[Pre-discharge Patients 12](#_Toc74921880)

[Provision of Information and Acquiring Consent 12](#_Toc74921881)

[Procedures 13](#_Toc74921882)

[Intervention Arm – Eliminating and/or Reducing Mobilisation Alarms 13](#_Toc74921883)

[Control Arm – Observing Mobilisation Alarms and Provision of Evidence 14](#_Toc74921884)

[Staff Workshop and Interviews 15](#_Toc74921885)

[Pre-discharge Patients 16](#_Toc74921886)

[Outcome Measures and Data Collection 16](#_Toc74921887)

[Data Management 18](#_Toc74921888)

[Data Coding 18](#_Toc74921889)

[Data Transfer 19](#_Toc74921890)

[Data Storage 19](#_Toc74921891)

[Data Access 20](#_Toc74921892)

[Data Use 20](#_Toc74921893)

[Data Archive 20](#_Toc74921894)

[Data Analysis 20](#_Toc74921895)

[Data Linkage 21](#_Toc74921896)

[Results, Outcomes and Future Plans 23](#_Toc74921897)

[References 24](#_Toc74921898)

# Background

Mobilisation alarms are a staple in hospitals as part of the falls prevention management repertoire, they can take up to 11% of all falls prevention management costs (Mitchell et al., 2018). However, a 2018 Cochrane review conducted a meta-analysis that found “uncertain evidence of the effects of bed and chair sensor alarms in hospitals” (Cameron et al., 2018). The review’s statistical findings suggested that there may be benefits the use of alarms in reducing falls rate, but this is not definitive. This is a problem as a wide-spread practice with uncertain effectiveness can use up resources that would be better spent on known effective practices or developing innovative approaches. Based on a recent study conducted by Monash University in 6 Victorian health services, the cost of purchasing, application, and staffing mobilisation alarms consume over 4.5 million Australian Dollars per annum (Mitchell et al., 2018). Closer examination of the use of these alarms has also highlighted impacts on nursing staff and patients. Nursing staff have reported to experience anxiety related to the use of alarms, and have to disrupt important tasks at hand to respond to alarms (King, Pecanac, Krupp, Liebzeit, & Mahoney, 2018), which may lead to detrimental errors in delivery care (Westbrook, Woods, Rob, Dunsmuir, & Day, 2010) and abandonment of tasks (Westbrook, Coiera, et al., 2010). Although not a physical restraint, the alarms have been cautioned by experts as a form of restraint on patients (Inouye, Brown, & Tinetti, 2009), which not only do not reduce falls but can also lead to other adverse outcomes (Lach, Leach, & Butcher, 2016). **Disinvestment** was proposed as a method to address the uncertainties in falls prevention strategies (Mitchell et al., 2018).

The term “***disinvestment***” is defined as the ***displacement of non–cost-effective approaches for resource reinvestment or reallocation*** (Joshi, Stahnisch, & Noseworthy, 2009). To maximise efficiency and effectiveness of research, we propose the disinvestment process be integrated with a stepped-wedge design, where wards begin with usual practice and are phased into the disinvestment condition over time in random order; non-inferiority margin stopping rules will be set to ensure maximum safety. This approach allows for comparisons within and between wards across time, and gives participating health professionals and service managers to contribute to the determination of stopping-rules during the trial. In doing so, the proposed study will produce robust findings of the effectiveness of mobilisation alarms, the effectiveness of the disinvestment process, and the economic efficiency of the process on mobilisation alarms. The outcomes will also go on to provide an efficient model for health service quality assurance projects in Australia and world-wide.

The four stages of disinvestment are 1) identification of low-value practices, 2) facilitate the disinvestment process, 3) evaluate outcomes, and 4) sustain the disinvestment (2). As the potential low-value practice has already been identified as mobilisation alarms. This proposal will detail steps 2 to 4 in a trial with a stepped-wedge design.

## Research Aims

1. Examine the effectiveness of a disinvestment strategy implemented through a randomised stepped-wedge design in facilitating practice change in mobilisation alarm use.
2. Examine the sustainability of disinvestment outcomes.
3. Understand stakeholder acceptability, the mechanisms of impact and sustainability of the disinvestment process.
4. Examine the economic impact of disinvestment in mobilisation alarm use.

## Expected Outcomes

Based on pilot study results (Brusco et al., 2021), we hypothesise that removal and/or reduced use of mobilisation alarms can be achieved for hospital wards that understand and accept the evidence and the study’s conditions. During the disinvestment period, there will not be an increase in the number of falls or falls with adverse events in hospital wards. Sustainability of the practice following the end of the disinvestment period is unknown, and the findings of the proposed study will shed light on this matter.

It is anticipated that a range of responses will be gathered from stakeholders with regards to the application of disinvestment in a hospital setting. These responses may range from positive to negative, and the insight provided by these responses will identify health professionals' knowledge, beliefs, and attitudes that can facilitate or be barriers to the disinvestment process.

# Project Design

## Research Project Setting

This project will be carried out in 36 wards across at least 7 public and private health services in Victoria, with a portion of the data collection to be potentially completed online in compliance with COVID-safe guidelines. The process of disinvesting from mobilisation alarms will be conducted in acute and sub-acute hospital wards that meet inclusion criteria, with data to be collected by an onsite staff on a regular basis. Workshops and interviews will be conducted with staff from participating wards at 4 time points during the duration of the study; these will have the options to be completed online via the zoom.us platform for interviews and video sharing for workshops to minimise COVID-19 infection risk.

## Methodological Approach

This will be a concurrent, nested, randomised stepped-wedge study with a mixed methods and non-inferiority design (Figure 1). Half of the wards (n=18) will be assigned to the intervention arm, the other half to the control arm. All wards in the intervention arm will begin under the same “usual care” condition, pairs of wards will begin the disinvestment condition at different time points, which will allow for comparisons of the ward with itself under different conditions: a) usual care of utilising the mobilisation alarms, and b) removing or reducing its use. Randomisation will determine whether each ward undergoes a removed or reduced mobilisation alarm condition, and the timing at which they phase into this condition. All wards in the control arm will be observed during the same time period, with no disinvestment process in place, but will receive the evidence arising from the intervention arm.

The mixed methods design combines quantitative and qualitative methodologies for the data collected and their analysis. Quantitative methods will be used to collect data for the rate of use of mobilisation alarms and other falls prevention strategies, resources allocated to the implementation of these strategies, and the clinical outcomes for participating wards. A narrative inquiry approach will be used to collect data regarding stakeholder acceptability of the disinvestment process; stakeholders will include health professionals and patients.

This study will also be a non-inferiority trial, this means we will try to prove that the removal/reduction of mobilisation alarms will not result in worse clinical outcomes. Therefore, a non-inferiority margin will be agreed upon with all health services in the intervention arm. The margin will be a specific rate of falls (averaged across all health services) that is just below the usual average rate of falls that is deemed unacceptable. For example, if the average number of falls that occur across Victoria is Y per week, then 150% more than Y falls per week while alarms are reduced/removed will see a stop to the entire project.

## Participants

Based on sample size calculations, a reduction in the proportion of wards with high bed alarm use requires 15 wards to achieve 80% power with a two-tailed alpha of 0.05. The nesting of wards within health services (3 wards per service on average) requires adjustment for this design effect. A pilot study we conducted in Victoria (Brusco et al., 2021) identified an intra-class correlation of bed alarm use of 0.10, indicating that we will require 18 wards nested within 6 health services. We will recruit these 18 wards across 6 public or private metropolitan health services in Australia. The control arm will be recruited from different health services, and will also consist of 18 wards to match the intervention arm. The same inclusion and exclusion criteria will be applied to both the intervention arm and control arm.

Inclusion criteria: wards with a mobilisation alarm use rate of at least 3% will be eligible for participation in the study. This will be determined by daily audits over a 2-week period.

Exclusion criteria: paediatric, emergency, intensive care, and mental health wards, as well as residential aged care facilities within health services will not be recruited for this study.

In the intervention arm, 2 groups of individual participants will be recruited:

1. Up to 5 staff members from each participating ward and the falls prevention portfolio manager will be recruited to provide feedback on their attitudes and perceptions of falls prevention, mobilisation alarms, and this project. This will be conducted at 4 times points during the project.
2. A random selection of patients who are 36 hours prior to discharge in each ward will be recruited to complete 2 brief questionnaires to provide feedback on their hospital stay. This will be conducted on a weekly basis.

In the control arm, 1 group of individual participants will be recruited:

1. Up to 5 staff members from each participating ward and the falls prevention portfolio manager will be recruited to provide feedback on their attitudes and perceptions of falls prevention, mobilisation alarms, and this project. This will be conducted at 3 times points during the project.

## Participant Recruitment and Time Frame

### Health Services

The project executive team consists of members from 6 health services in Melbourne, Victoria: Epworth, Eastern, Monash, Peninsula, Alfred, and Cabrini. These health services will be recruited for the intervention arm. Representatives of each health service will work together with the health service to identify the wards that would be suitable for this project. The project manager, Dr. Debbie Pu, will liaise with site representatives to prepare and submit the documents needed for this process at each site in addition to the human research ethics committee (HREC) approval and site specific assessment (SSA) for each health service.

Following HREC approval, the project team will also recruit health services for the control arm via advertising and engagement of the falls prevention teams at various health services in Victoria. The project manager will engage with potential health services via publicly available phone numbers and email addresses. When the appropriate personnel has been engaged, a 1-page document outlining the project’s design and the control arm’s involvement will be emailed. The project manager will follow-up this email with a phone call in one week’s time. Health services that consent to participating in the study will liaise with the project manager to acquire SSA.

### Hospital Wards

Following approvals, we will ask site representatives to nominate wards that match the inclusion and exclusion criteria. For each nominated ward, a 2-week audit will be conducted to determine eventual eligibility: daily audits of mobilisation alarm use rate will be tallied to confirm the minimum 3% use rate. We anticipate approximately 1-2 months to screen the wards and recruit staff on the wards to participate in the qualitative component of the project.

### Hospital Staff

Wards that are deemed eligible for the study will be informed of the project by the site liaison at each health service during a weekly staff meeting following site special approvals are granted. The site liaison will be named principal investigator for each site, they will be named as the primary contact person for the study at their health services and their contact details (phone number and email) will be available on the participant information and consent forms (PICF). Staff, including the falls prevention portfolio manager at the health service, will be given a copy of the participant information and consent form (PICF) outlining their expected involvement. The site liaison will follow up at the next weekly meeting and obtain signed consent forms from up to 5 ward staff who are interested. Staff may also contact the site liaison via the provided phone number or email to express their interest to participate in the study. A copy of the PICF with contacts details for queries and complaints will be provided at the end of participation.

### Pre-discharge Patients

Recruitment of patients who are 36 hours pre-discharge will be conducted in the intervention arm by the onsite research assistant on a weekly basis during the study period. A ward will be randomly chosen each week, hospital records will be used to identify patients in participating wards who will be discharged in the next 36 hours. Research assistants will approach these patients, explain the project, and ask for consent to participate. Patients will be given 5 minutes to consider their participation. Participants will be asked to sign a PICF before any data is collected. A copy of the PICF with contacts details for queries and complaints will be provided at the end of participation. This process will begin as soon as the study begins and continue until all wards have undergone mobilisation alarm reduction/removal and the trial ends.

## Provision of Information and Acquiring Consent

Participant Information Sheet/Consent Forms (PICF) will be provided to the two groups of individual participants we will be collecting data from:

1. Hospital staff who will be part of the project before the start of the stepped-wedge trial. Following HREC and SSA approval, site liaisons will help to disseminate project information and PICFs to staff on participating wards. They will be given 1 week to consider the information if needed.
2. Patients who are 36 hours prior to discharge in participating wards. These patients will be provided with a PICF that explains the background of the project and what they would be expected to do if they decide to take part. They will be given 5 minutes to consider the information if needed.

All individual participants will be informed of the option to withdraw at any time without consequences. A study withdrawal form will be provided to ward staff who wish to withdraw. If at any time during the study period there are queries and concerns, they will be advised to direct these to the site specific principal investor, or overall project manager (Dr. Debbie Pu), or the chief investigator of the project (Prof. Terrence Haines). They may also direct these to the Monash University Human Research Ethics Committee. This information will be available in the PICFs and verbally highlighted by the researcher or research assistant who obtains consent from participants.

## Procedures

### Intervention Arm – Eliminating and/or Reducing Mobilisation Alarms

To assess the effectiveness of mobilisation alarms, their use will be eliminated or reduced in tertiary hospital wards. The elimination and reduction of alarms will be the intervention. A stepped-wedge design will be used for intervention. Pairs of wards (clusters) will be randomised into either eliminated (0%) or reduced (<3%) mobilisation alarm use, they will then enter the intervention conditions in sequential order month by month, until all wards are under the intervention conditions (Figure 1). Baseline assessments will be conducted over a 1-month before the first cluster begins intervention, the entire intervention period will last 9 months, with different clusters under intervention for different lengths of time. One week prior to each cluster beginning intervention, the ward staff will be interviewed for their perception of the study. Follow-up assessments will be conducted at 1 month and 13 months after the end of intervention. Participating health services and wards will be required to comply with the intervention condition they are assigned to, weekly auditing of mobilisation alarm use rate will be conducted to ensure they are eliminated and/or reduced as required. During the period of intervention, falls rates and other related outcomes will be collected on a weekly basis by the site research assistant (Table 1). The rate of newly diagnosed COVID cases will also be documented each week, as this continues to be an ongoing concern in hospitals and may interfere with daily procedures that affect falls management in wards.


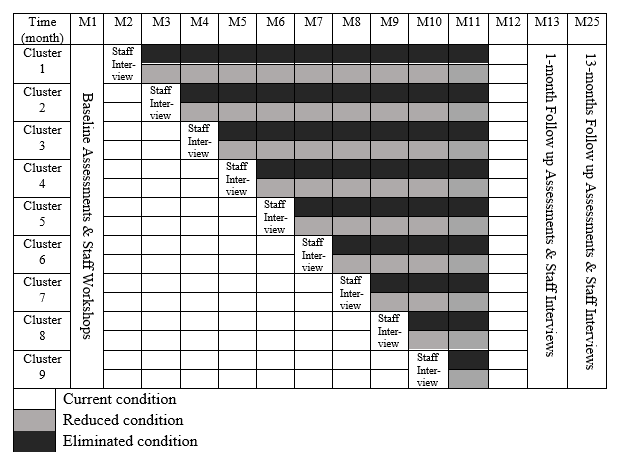


**Figure 1. Stepped-wedge design proposed in the current study.**

### Control Arm – Observing Mobilisation Alarms and Provision of Evidence

The control arm will follow the same schedule as the intervention arm, but with no disinvestment interventions. Data will only be collected at 3 time points: baseline, 1-month follow up, and 12-month follow up (Figure 2).


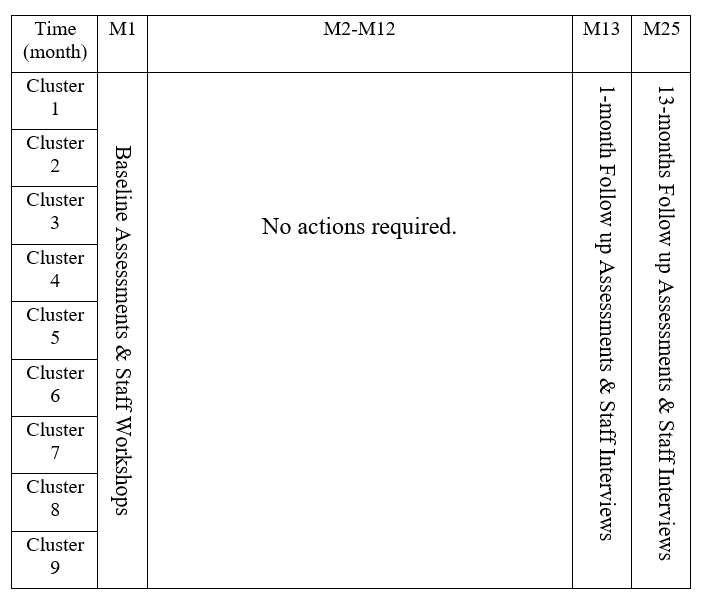


**Figure 2. Data collection schedule for the control arm.**

### Staff Workshop and Interviews

Around 5 staff from each participating ward and the falls prevention manager at each health service will be interviewed at 4 time-points:

1. Baseline (intervention and control arms) – a workshop describing the most up-to-date research evidence for mobilisation alarms will be given in the form of PowerPoint presentation and infographic video. Following this, staff will be asked about their attitudes towards mobilisation alarms and participation in the study.
2. 1 week before intervention condition begins (intervention arm only) – staff will be asked again about their attitude towards participation in the study.
3. 1 month following the end of intervention (intervention and control arms) – staff will be informed of the findings of the study, and will be asked about their experience in participating in the study and if and how their attitudes towards mobilisation alarms have changed.
4. 12 months following the end of intervention (intervention and control arms) – staff will be asked about their attitude towards mobilisation alarms and if there have been any practice changes for their use.
5. Practice implementation questionnaire (intervention and control) – the falls prevention portfolio will be asked to complete a brief rating scale (~5 minutes) about the implementation of evidence-based falls prevention practice at their health service at each data collection point described above.

The workshop and interviews will have the option of being conducted over online platforms to avoid in-person researcher visits to health services. The synchronous option will use the zoom.us platform for the presentation and to conduct group interviews with the participating staff. The interview will be voice-recorded for later analysis. The asynchronous option will involve uploading the presentation to an online video-hosting platform, which will allow participating staff access in their own time, and feedback from staff will be sought via online questionnaires/surveys.

### Pre-discharge Patients

A random sample of patients across participating wards in the intervention arm will be requested to complete two surveys at 36 hours prior to their discharge, this will be conducted throughout the duration of the study. One survey will be the question “Overall, how would you rate the care you received while in hospital?”, response categories for this item are Very Good, Good, Average, Poor, Very Poor. The second survey will be the 10-question Pittsburgh Sleep Quality Index (Carpenter & Andrykowski, 1998).

### Health Service Implementation Strategies

The health services are anticipated to carry out support strategies for the implementation of alarm use change in this study. For the purpose of understanding intervention fidelity, members of the research team who will be visiting study sites and contributing to the coordination of study activities will take field notes at strategy meetings.

### Falls Prevention Guideline Documents

The official health service falls prevention and falls alarm use documents may or may not change following the trial and provision of evidence from the trial. Therefore, official guidelines and documents will be accessed from each health service for the periods before the trial (prior to April 2023), immediately after the trial (anticipated February 2024), and at the 12-month post-trial follow-up (anticipated February 2025). These documents are accessible via the staff intranet, site investigators will be responsible for accessing them.

## Outcome Measures and Data Collection

| **Table 1. Outcome measures and their data collection approach in the intervention arm.** | | |
| --- | --- | --- |
| **Outcome** | **Outcome Type** | **Data Collection Approach(es)** |
| Rate of Falls | Primary Clinical Outcome | Extraction from hospital records  Interview with nurse unit managers (NUM) |
| Rate of roll-outs from low-low beds | Secondary Clinical Outcome | Extraction from hospital records  Interview with nurse unit managers (NUM) |
| Rate of falls-related injuries | Secondary Clinical Outcome | Extraction from hospital records |
| Patient satisfaction with care –  Victorian Patient Satisfaction Survey (Draper, Cohen, & Buchan, 2001) | Secondary Clinical Outcome | Interview with patient sub-sample |
| Patient sleep quality –  Pittsburgh Sleep Quality Index(Carpenter & Andrykowski, 1998) | Secondary Clinical Outcome | Interview with patient sub-sample |
| Rate of health service code greys | Secondary Outcome - Non-falls Related Adverse Events | Extraction from hospital records |
| Rate of newly developed pressure injuries (since admission to ward) | Secondary Outcome - Non-falls Related Adverse Events | Extraction from hospital records |
| Rate of medication error | Secondary Outcome - Non-falls Related Adverse Events | Extraction from hospital records |
| Rate of hospital readmission within 30 days | Secondary Outcome - Non-falls Related Adverse Events | Extraction from hospital records |
| Proportion of patients with mobilisation alarms | Secondary Outcome – Intervention Fidelity | Direct observations of ward beds |
| Rate of use of “other” confounding falls-prevention interventions | Secondary Outcome – Intervention Contamination | Direct observations of ward beds |
| Casemix payments to hospital | Secondary Intervention Outcomes | Extraction from hospital records |
| Procedures subsequent to falls-related injury | Secondary Intervention Outcomes | Extraction from hospital records |
| Staff attitudes to involvement in trial | Other Secondary Outcomes | Interview with NUMs and ward staff at the start of the trial |
| Staff concerns regarding transition from Current condition to either Reduced or Eliminated conditions | Other Secondary Outcomes | Interview with NUMs and ward staff prior to transition |
| Staff experiences of transition and intervention | Other Secondary Outcomes | Interviews with NUMs and ward staff at 1-month and 12-months post-trial follow-ups |
| Knowledge translation and implementation of evidence-based practices (Stages of healthcare implementation rating scale, unpublished) | Other Secondary Outcomes | Falls prevention portfolio manager completion of rating scale |

# Data Management

## Data Coding

Data will take the form of hard copies of surveys and written notes made during interviews, and soft copies of electronic records of falls and mobilisation alarm-related data on tablet computers. Individuals that provide data will be assigned a code on documents to protect their privacy, for example P123 for patients who complete surveys before discharge, CL123 for staff who participate in interviews. Identifying information such as name and diagnosis will not be included in the data.

## Data Transfer

PICFs will be stored locally (at the site) for all participants. Both hard and soft copy data will be collected from the RA each week by the project manager (Dr. Debbie Pu). Cursory data checking will be done by the project manager to ensure no missing data. Hard copy data will be sealed in opaque envelopes with the RA and project manager as witness until they are opened for data entry at Monash University. Soft copy data will be emailed to a project-specific Monash University email inbox, in a password-encrypted folder. The password will be generated by a random password generator, a new password will be generated every month. The data transfer will be conducted on the premises of each health services.

## Data Storage

Hard copies (if any) of data will be stored in a locked cabinet at in Building G, Peninsula Campus, Monash University. Copies of the key will be held by the project manager (Dr. Debbie Pu) and chief investigator (Prof. Terrance Haines) only. Hard copies will be destroyed 7 years after the completion of the study.

Electronic copies of data will be stored as two versions:

1) one in the project-specific Monash University inbox (sent by research assistants at each site following weekly data collection), which requires 2-factor authentication log in by a research team member. This data will be deleted ~~5~~7 years after the completion of the study.

2) another copy will be stored on the platform LabArchives in a Monash University affiliated account, which will archive the data. This account will require a Monash University email to log in, with 2-factor authentication. This will not be deleted, and will be archived in accordance with Monash University data archiving guidelines.

## Data Access

Any access to the data following storage will need to be informed either the project manager or chief investigator, with documentation of each personnel who requested data, the data they requested access to, and reason for access.

## Data Use

The data collected for this project is for the sole purpose of research. Analysis and interpretation of the data will be for the primary purpose of addressing the research objectives raised in this proposal. If data is requested for the purpose of other research objectives, the chief investigator will consider each request on its individual merit.

## Data Archive

The data for this project will be archived accordingly to the above described steps following the conclusion of the project.

## Data Analysis

A reduction in the proportion of wards with high bed alarm use from 100% to 50% requires 15 wards per group to achieve 80% power with a two-tailed alpha of 0.05. The nesting of wards within health services (3 wards per service on average) requires adjustment for this design effect. Our disinvestment pilot study (Brusco et al., 2021) identified an intra-class correlation (ICC) of bed alarm use of 0.10, indicating we require 18 wards per group nested within the 6 health services. With this sample size, the intervention will have 86% power to detect a 10% relative difference in fall rates with a mean baseline of 8 falls (standard deviation=4) per month per ward, and an ICC of fall rates between wards of 0.63 based upon 1000 bootstrap power analysis simulations (Stata MP version 14.0).

Participating wards will be matched by type (acute, subacute) into clusters. Wards in each cluster will undergo the same period of intervention, allowing for comparison of varying degrees of disinvestment. Falls and alarm use rate across time will allow for the examination of the feasibility of disinvesting mobilisation alarms and its effects on falls rate and whether these effects can be sustained.

The content of post-workshop interviews and recordings of interviews with health service staff will be thematically analysed. The software NVivo will be used to aid in the coding, linking, categorising, and theme identification. Interview transcripts will be analysed independently by at least two researchers.

The cost-benefit analysis is calculated by summing the costs of the benefits and then subtracting the costs of implementation. The primary cost-benefit analysis will include the direct and indirect benefits, the secondary cost-benefit analysis will only include the direct benefits. Data will be analysed and presented as a total across the six participating health services as well as individually for each health service. Reporting for each health service individually will provide data on the range of implementation costs borne by the different health services. Average cost per health service will be reported as a mean value with a standard deviation, and the costs for the different health services will be compared with an independent t-test, where p<0.05 will be used to determine statistical significance.

## Data Linkage

Data linking will not be required for most of the primary outcomes of this project. Interviews with health professionals in participating wards will use coding to identify the same individual across different interviews. Each interviewee will be assigned a code, and the same researcher will be conducting interviews throughout the duration of the study. NVivo will assist in data linking for recordings of interviews.

# Results, Outcomes and Future Plans

Data will be regularly audited by an independent monitoring committee, comprising of two research team members (Prof. Terrence Haines and Dr. Kelly-Ann Bowles) and one representative from each of the intervention arm health services in the study. This will ensure that each health service has access to study results as they arise. Each ward that participates in the study will be debriefed at the end of the intervention period and after the conclusion of all data collection regarding overall findings.

A number of scientific papers will be written to address each of the research aims already presented. These will be submitted to high-impact peer-reviewed journals in the area of health and medicine for publication.

The data obtained from this study may be used for research purposes not addressed in the current proposal. This study will be a large-scale multi-centre trial, therefore the results may be used as normative or reference data for other studies in the disciplines of health and falls prevention.

# References

Brusco, N., Hutchinson, A., Mitchell, D., Jellett, J., Boyd, L., Webb-St Mart, M., . . . Botti, M. (2021). Mobilisation alarm triggers, response times and utilisation before and after the introduction of policy for alarm reduction or elimination: A descriptive and comparative analysis. *International Journal of Nursing Studies, 117*, 103769.

Cameron, I. D., Dyer, S. M., Panagoda, C. E., Murray, G. R., Hill, K. D., Cumming, R. G., & Kerse, N. (2018). Interventions for preventing falls in older people in care facilities and hospitals. *Cochrane database of systematic reviews*(9).

Carpenter, J. S., & Andrykowski, M. A. (1998). Psychometric evaluation of the Pittsburgh sleep quality index. *Journal of psychosomatic research, 45*(1), 5-13.

Draper, M., Cohen, P., & Buchan, H. (2001). Seeking consumer views: what use are results of hospital patient satisfaction surveys? *International journal for quality in health care, 13*(6), 463-468.

Inouye, S. K., Brown, C. J., & Tinetti, M. E. (2009). Medicare nonpayment, hospital falls, and unintended consequences. *New England Journal of Medicine, 360*(23), 2390.

Joshi, N. P., Stahnisch, F. W., & Noseworthy, T. W. (2009). Reassessment of health technologies: obsolescence and waste.

King, B., Pecanac, K., Krupp, A., Liebzeit, D., & Mahoney, J. (2018). Impact of fall prevention on nurses and care of fall risk patients. *The Gerontologist, 58*(2), 331-340.

Lach, H. W., Leach, K. M., & Butcher, H. K. (2016). Changing the practice of physical restraint use in acute care. *Journal of gerontological nursing, 42*(2), 17-26.

Mitchell, D., Raymond, M., Jellett, J., Webb-St Mart, M., Boyd, L., Botti, M., . . . Haines, T. (2018). Where are falls prevention resources allocated by hospitals and what do they cost? A cross sectional survey using semi-structured interviews of key informants at six Australian health services. *International Journal of Nursing Studies, 86*, 52-59.

Westbrook, J. I., Coiera, E., Dunsmuir, W. T., Brown, B. M., Kelk, N., Paoloni, R., & Tran, C. (2010). The impact of interruptions on clinical task completion. *BMJ Quality & Safety, 19*(4), 284-289.

Westbrook, J. I., Woods, A., Rob, M. I., Dunsmuir, W. T., & Day, R. O. (2010). Association of interruptions with an increased risk and severity of medication administration errors. *Archives of Internal medicine, 170*(8), 683-690.
