# Supplementary material for: Does the site of research evidence generation impact on its translation to clinical practice? A protocol paper
Source: PLoS One. 2024 Dec 13;19(12):e0314956. doi: 10.1371/journal.pone.0314956 (PMC11643258; doi:10.1371/journal.pone.0314956)
Supplement: Attachment — Submitted filename: Response to review 1_16April24.docx [file pone.0314956.s005.docx]

Thank you to the editorial team and reviewers for providing feedback on our protocol manuscript. We have considered all the suggestions and made amendments where we thought appropriate, and have provided explanations for comments where we did not think amendments were necessary. Our responses are underlined below for your convenience:

**Editorial comments:**

1. Please ensure that your manuscript meets PLOS ONE's style requirements, including those for file naming.

Headings and in-text references have been amended in accordance to the PLOS ONE style templates provided.

2. Please state what role the funders took in the study.  If the funders had no role, please state: ""The funders had no role in study design, data collection and analysis, decision to publish, or preparation of the manuscript."" 
If this statement is not correct you must amend it as needed. 
Please include this amended Role of Funder statement in your cover letter; we will change the online submission form on your behalf.

The role of the funder was stated in the paragraph immediately following the trial funding statement on page 18 of the originally submitted manuscript. We have now edited this statement to match the wording suggested, and have also added this statement to the revised cover letter.

3. We note that you have provided funding information that is not currently declared in your Funding Statement. However, funding information should not appear in the Acknowledgments section or other areas of your manuscript. We will only publish funding information present in the Funding Statement section of the online submission form. 
Please remove any funding-related text from the manuscript and let us know how you would like to update your Funding Statement. Currently, your Funding Statement reads as follows: 
"Trial funding:
National Health and Medical Research Council, Australian Government. Grant number: APP1186185"

Funding information has now been removed from the acknowledgement section. We will be keeping the funding statement the same.

4. Thank you for stating the following in the Competing Interests section: 
"TPH has provided expert witness testimony on the subject of prevention of falls in hospitals for K&L Gates Law Firm and Minter Ellison Law Firm."

Please confirm that this does not alter your adherence to all PLOS ONE policies on sharing data and materials, by including the following statement: ""This does not alter our adherence to PLOS ONE policies on sharing data and materials.” (as detailed online in our guide for authors [http://journals.plos.org/plosone/s/competing-interests](about:blank)).  If there are restrictions on sharing of data and/or materials, please state these. Please note that we cannot proceed with consideration of your article until this information has been declared.

Please include your updated Competing Interests statement in your cover letter; we will change the online submission form on your behalf.

This competing interest statement does not alter our adherence to all PLOS ONE policies on sharing data and materials. We have added the competing interest and the additional detail in the revised cover letter.

5. Please include a separate caption for each figure in your manuscript.

Caption for figure 1 now amended on page 7 of manuscript.

6. Please include captions for your Supporting Information files at the end of your manuscript, and update any in-text citations to match accordingly. Please see our Supporting Information guidelines for more information: [http://journals.plos.org/plosone/s/supporting-information](about:blank).

Supplementary materials renamed and caption added on page 18 of the manuscript.

**Reviewer 1 Comments:**

Since this manuscript is a protocol, it is better to change the title of this manuscript to match the original protocol. The current title of this manuscript sounds like a manuscript reporting the research results.

The current title is “Does the site of research evidence generation impact on its translation to clinical practice? *A protocol paper*”. We are not sure what the reviewer is referring to by “original protocol”. If this means the already published protocol that is related to this one, the two are different studies with different designs and the titles for each were chosen with care to match the content (Please refer to paragraph 1 in the discussion section). We are open to other suggestions for the title if the reviewer has any specific suggestions.

The section of Design was too brief to inform the proposed design. Sample size has a similar issue.

The paragraph under the subheading of “Design” was written to provide a description of the overall study design before we described the study in more detailed components in subsequent sections and subheading. Figure 1 was also added to this section to provide a visual overview. We have decided not to add more details to this section as these will be provided later in the manuscript, we hope the reviewer understands.

The sample size description was brief as the 18 wards to be recruited for this study are to match the 18 wards already recruited for the “evidence generation” arm, which was based on power calculations. We have added a clarification of this to the section, which now reads:

“This study will involve 36 hospital wards. Eighteen wards have been recruited for the disinvestment trial that make up the “evidence generation” arm, this was based on power calculations described in the protocol paper that described the disinvestment trial,[16] therefore a matching number of 18 wards will be recruited for the “external impact” arm.”

What’s the difference from the previous protocol published in 2021? Was the control arm added to the previous protocol? The 1st paragraph of discussion does not make those questions clear.

The two protocols describe two related but different studies. The previous protocol details a clinical trial that will generate the research evidence that will be used as the “intervention” for the study described in this current protocol. The previous protocol did not mention the control arm described in the current protocol. We have attempted to delineate the two in this paragraph, but given your comments we have made minor edits to make this more explicit. The final sentence of this paragraph now reads:

“The disinvestment trial focuses on the effectiveness of mobilisation alarms for falls prevention, and the previously protocol [16] described the changes in alarm use across the recruited wards; while the current protocol describes a study that will use the mobilisation alarm context to evaluate the effect of -site of evidence generation on the implementation of evidence into practice, including local policy change.”

The English needs professional edits. E.g. “New staff who have no attended previous interviews will be asked to provide informed and written consent before been interviewed.”

Apologies for the typographic errors in the manuscript, we have proof read more thoroughly for the revised submission.

It is good to compare before and after intervention in evidence generation arm. It might not be comparable between evidence generation arm and external impact arm in fall rate since the wards recruited for each group are from different services and the risk of fall might be different a lot.

As the evidence generation arm is a different study, the analyses for that study was not discussed in this protocol. Falls rate is not an outcome measure for this study, as we are measuring adoption of evidence for fall prevention rather than the effects of any fall prevention strategies adopted. We hope our previous response to the questions about the differences between the already published protocol for the evidence generation arm and this protocol’s external impact arm is able to address this comment as well.

The format of this manuscript sounds for reporting results from a study instead of a protocol.

Our manuscript format is in accordance with PLOS ONE’s criteria for study protocols: [Submission Guidelines | PLOS ONE](about:blank#loc-study-protocols)

**Reviewer 2 comments:**

In general, thank you for your encouraging comments and well wishing for our study.

I realise more information is likely available in the first protocol paper but nevertheless, I think a more detailed description of how and why the 18 wards were chosen and on what criteria were they matched is warranted here. This is critical for establishing the efficacy of the implementation. Also, the characteristics and nature of these wards perhaps are likely to be more deterministic as opposed to whether they were involved or not. Establishing any kind of causality without measuring a range of other characteristics and controlling for them will be difficult. This should be acknowledged as a limitation of the trial. For example, any number of implementation predictors (like leadership, organisational culture, ward type, patient mix etc) could predict uptake. Also, can you clarify whether the matched wards are within the same hospital site?

The wards were not planned to be a 1 on 1 match, but we did try to recruit all ward types and managed to balance acute versus subacute wards. Two wards are in the same two hospitals as two wards in the evidence general arm. This has been added to page 9 of the manuscript:

“There were no minimum requirements for bed numbers and alarm use rate for this arm. Both acute and subacute wards were recruited: 11 acute and 7 sub-acute wards were recruited for each arm. As much as possible, we tried to avoid recruiting wards that would be in the same hospital as wards in the evidence generation arm, although they could still be in the same health service. The final 18 wards recruited included two wards that shared a hospital site with two wards in the external impact arm, which meant that two hospitals hosted both evidence generation arm and external impact arm wards.”

Your comments about acknowledging other potential contributors to the wards implementing something is noted. We have added a “limitations” section at the end of the discussion to acknowledge this.

This point also links to my second observation is on the lack of acknowledgement of the factors that predict or are related to implementation success as acknowledged and studied in a myriad of implementation science frameworks (e.g. CFIR, PARIHS etc). Are you employing an implementation science framework for examining the factors that drive success in the disinvestment trial? If not, what was the reason for this choice?

We had planned to adopt a realist evaluation approach in the original study designs (detailed in the grant proposal document attached as part of the submission), this has now been added to the manuscript at the end of page 12 and flowing onto the start of page 13:

“We will follow a narrative inquiry approach consistent with the realist evaluation framework seeking to understand what works, for who, when, and in what circumstances.”

There are a few typographical errors throughout (missing t and s) so a good proofread would be advisable.

Apologies for the typographic errors in the manuscript, we have proofread more thoroughly for the revised submission.
