# Supplementary material for: Does the site of research evidence generation impact on its translation to clinical practice? A protocol paper
Source: PLoS One. 2024 Dec 13;19(12):e0314956. doi: 10.1371/journal.pone.0314956 (PMC11643258; doi:10.1371/journal.pone.0314956)
Supplement: Attachment — Submitted filename: Response to review 2_20Aug24.docx [file pone.0314956.s006.docx]

Thank you to the reviewers for their thoughtful feedback. Our responses are underlined below for your convenience:

**Reviewer 1 Comments:**

- In quantitative analysis, please make it clear what outcomes will be analyzed with repeated measures ANOVA?

Upon further discussion, we have amended the analysis approach from repeated measures ANOVA to a linear mixed model analysis. The quantitative analysis section now reads:

“The rate of mobilisation alarm usage will be calculated across time (T1, T2, T3) as proportion of patients with alarms during the 2-week audit periods. Linear mixed model analysis will be used to examine the difference in change (time by group interaction) in the proportion of audit days where the ward uses alarms in alignment with the recommendation between the wards that are involved in the disinvestment trial (evidence generation arm) and those that were not (external impact arm).”

- The sample size of 18 wards was determined for evidence-generation arm based on power analysis. But for the sample size for the “external impact” arm, it is lack of power analysis and no considerations for the expected difference in comparing outcomes between two arms. It needs more thought or provide discussions for the limitations of simply using 18 wards each arm for two arms comparison.

The sample size section has been amended to demonstrate the power provided by the 18-ward sample size, it now reads:

“A sample size of 18 wards who have been directly involved in the disinvestment trial (evidence generation arm), and 18 who have not (external impact arm), each with 10 audit-days of data before the workshop (external impact arm) and before the trial commenced (evidence generation arm) and 10-audit-days of data 12-months after the workshop (both arms) creates a total of 720 measurement points containing binary (aligned with recommendation, not aligned with recommendation) data. Baseline data, using a threshold of >3% bed alarm use as a proxy for this recommendation, indicates a proportion of 0.80 audit days exceed this threshold, that there is a standard deviation of 0.37 and correlation across measures within wards of 0.78. These data were used in Monte Carlo simulation modelling (1000 simulations) to identify that this sample size provides >80% power to detect a 0.20 absolute reduction in the number of audit days where the threshold is exceeded assuming the control condition proportion remains at the same level. If the external impact arm has a 0.20 absolute reduction in this proportion, then our sample size will provide >80% power to detect a further drop of 0.23 in the intervention group beyond the control group reduction.”

**Reviewer 3 comments:**

The protocol is nicely written. You have addressed the concerns and queries of previous reviewer comments. I have no further comments to make

We appreciate the positive feedback, thank you for your time.
